# Fatty Acids and Their Metal Salts: A Review of Their Infrared Spectra in Light of Their Presence in Cultural Heritage

**DOI:** 10.3390/molecules26196005

**Published:** 2021-10-03

**Authors:** Anna Filopoulou, Sophia Vlachou, Stamatis C. Boyatzis

**Affiliations:** Department of Conservation of Antiquities and Works of Art, University of West Attica, 12243 Egaleo, Greece; annafilopoulou.s@gmail.com (A.F.); ca15011@uniwa.gr (S.V.)

**Keywords:** fatty acids, metal soaps, oil binder, archaeological organic remains, infrared spectroscopy

## Abstract

In a cultural heritage context, fatty acids are usually found as breakdown products of lipid-containing organic remains in archaeological findings, binders in aged oil paintings, and additives in modern art-related materials. They may further interact with the ionic environment transforming into metal soaps, a process that has been recognized as a threat in aged paintings but has received less attention in archaeological objects. The investigation of the above related categories of materials with infrared spectroscopy can provide an overall picture of the organic components’ identity and demonstrate their condition and prehistory. The capability of investigating and distinguishing fatty acids and their metal soaps through their rich infrared features, such as the acidic carbonyl, the carboxylate shifts, the variable splits of alkyl chain stretching, bending, twisting, wagging, and rocking vibrations, as well as the hydroxyl peak envelopes and acid dimer bands, allows for their direct detailed characterization. This paper reviews the infrared spectra of selected saturated fatty monoacids and diacids, and their corresponding sodium, calcium, and zinc salts and, supported by newly recorded data, highlights the significance of their spectroscopic features.

## 1. Introduction

Lipids (glycerol triesters), existing as organic residues in archaeological findings (such as pottery and metal vessels) or as binders in oil paintings, are prone to hydrolytic damage producing free fatty acids (FA) [1,2,3,4]. Furthermore, FA in aged oil paintings may further react with abundant pigment metal ions in their molecular vicinity to form fatty acid metal salts (FAMS, metal soaps) [5,6,7]. Similar reactions may occur with calcium and other metal ions in archaeological samples, although they have been investigated in relatively fewer circumstances [8,9,10].

The analytical investigation of organic archaeological residues has received attention due to the resilience of organic molecules, such as lipids and their breakdown products, which helps researchers access information on dietary habits, rituals, and other practices developed by cultures of the past [11,12,13,14,15]. For instance, extended research has been done in ancient unglazed pottery since they offer a convenient protective medium, though allowing for the intrusion of organic compounds, preserves said compounds from environmental degradation for millennia [16,17,18,19,20,21,22,23,24,25,26,27,28,29,30,31,32,33,34,35,36,37,38]. Relatively fewer cases have been reported in wooden containers such as coffins [39] or metal alloys such as copper [9,40]. Most of the above cases have been studied through gas chromatography combined either with mass spectrometry (GC-MS) [18,20,28,33,34,38,41,42,43] or isotope ratio mass spectrometry (GC-IRMS) [30,36,37,38,41]. This technique allows for the accurate quantification of analytes. For instance, the oil content was analyzed in ancient amphorae found in the Late Bronze Age shipwreck of Uluburun [26] and the site of Amarna (Egypt) [44], or shipwrecks dating from the First Punic War [45]. In addition, the profiling of the adsorbed oil gradient within the clay fabric of potsherds has been investigated [44].

The acquisition of infrared spectra significantly assists analytical efforts, such as the ones mentioned above. Although detailed infrared analysis, such as the chemical mapping of paint cross-sections has been successfully conducted [46,47,48], the technique has generally been restricted to simple ‘screening’ or ‘fingerprinting’ in organic residue analysis. Nevertheless, its value has been recognized for guiding further analytical efforts [31,49]. Infrared spectroscopic analysis of lipid-containing samples is a fast technique that generally demands minimal sample workup, which based on their molecular bonding [24,50,51,52], provides rich information on their chemical components [50,53,54,55], such as glycerol esters, fatty acids, and metal salts. However, interpretation of complex samples can be tedious and problematic since frequent band overlaps impose difficulties for a secure assignment [56,57,58,59,60,61,62]. Thus, a necessity emerges for the accurate identification of fatty components in samples characterizing their existing condition through close examination of their infrared spectra.

In addition, advanced technological capabilities have led to the development of reflection-based techniques in combination with microscopy [7,46,48,63,64,65,66,67,68], which vastly expanded the depth of information offered by the method.

In the field of art, infrared spectroscopy has played a significant role in identifying and studying the binding media in oil paintings [7,69,70], and possibly more importantly, the severe deterioration caused by the formation of metal soaps [7,47,69,71,72,73,74,75,76,77,78,79]. In archaeology, infrared spectroscopy has also played a decisive role in analyzing organic archaeological residues [9,11,32,39,40,79]. Identifying fatty substances through their infrared spectra can elucidate chemical transformations such as the mineralization of acidic organic components in aged samples. Notably, organic molecular markers that connect the current condition of the materials with their original one can assess the prehistory of cultural heritage objects and their possible uses in antiquity [9,11,25,32,39,66,80,81].

This paper reviews the infrared spectra of selected fatty monoacids, diacids, and their sodium, calcium, and zinc salts, and points out their detailed features for detecting these compounds directly in their samples and further assessing their structural condition. The rich information gained through the close inspection of spectroscopic features can help researchers benefit in their investigations to a level beyond typical infrared screening.

## 2. Results and Discussion

### 2.1. Molecular Structure Phenomena in Fatty Acids

**Figure 1:**
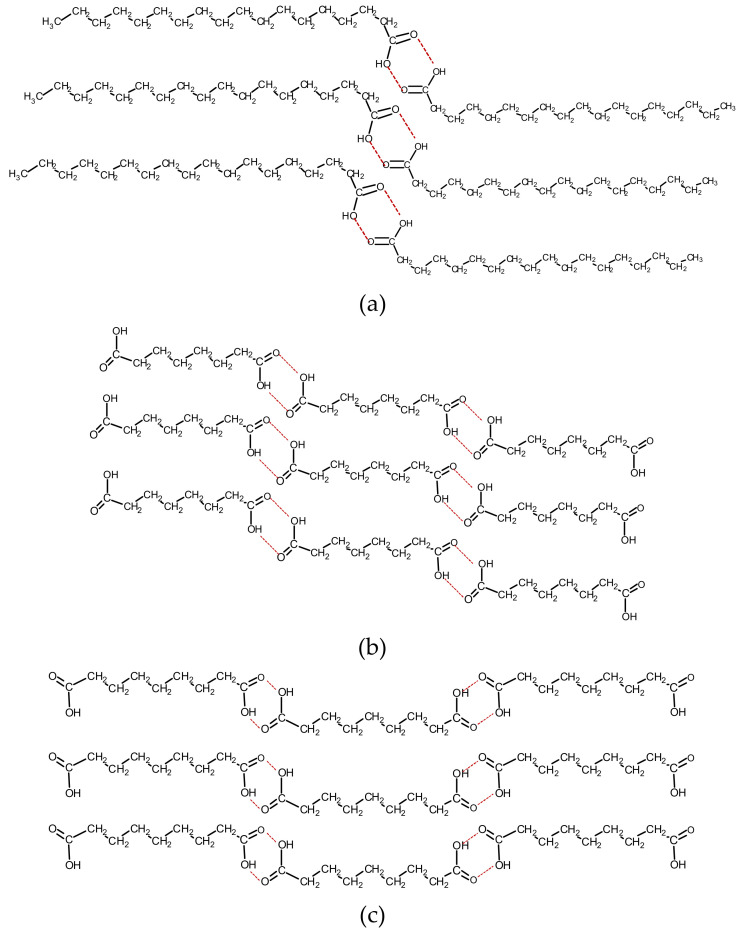
Molecular conformations of fatty acids. (**a**) Type B structure of stearic acid (adapted from [94]); (**b**) octanedioic acid (C8di) and (**c**) nonanedioic acid (C9di) (adapted from [96]).

The intermolecular attraction of acidic carbonyls due to hydrogen bonding induces a dimerization phenomenon where FA associate in a handshake mode forming head-to-head molecular pairs (or ‘dimers’) [82,83,84,85,86]. This was first shown experimentally by Pauling, followed by normal mode vibrational analysis [87,88]. In combination with hydrophobic alkyl chain associations, the above results in the bilayer coordination of fatty acid molecules described in Figure 1a [89,90,91]. Extensively studied through infrared spectroscopy, hydrogen bonding has been recognized and studied in alcohols and carboxylic acids since the 1930s [86,92,93,94,95,96]. As a concept, it gained profound attention in the second half of the previous century [87,88,95,97,98,99,100,101], while advanced computational capabilities during the last two decades have further led to elucidating intermolecular phenomena [102,103].

Carboxylic acids carry a hydrogen bond donor (the OH group) and a hydrogen bond acceptor (C=O) within the same unit and are, therefore, ideal molecules for studying the phenomenon. First, as a profound manifestation of the hydrogen bonding attraction, the hydroxyl stretch is considerably downshifted, compared to their ‘free’ state, and characteristically broadened [93,94,95,101]. Secondly, the dimerization phenomenon induces additional dimer infrared bands due to the impeded stretching of associated hydroxyls and carbonyls and restricted bending of the C‒O‒H group (see below). Moreover, intermolecular associations between long alkyl chains of FA induce crystal packing (Figure 1), affecting their melting points [104,105] and infrared spectra.

### 2.2. Infrared Spectra of Saturated Monoacids and Their Metal Salts

#### 2.2.1. Monoacids

The infrared spectra of saturated fatty acids (sFA) have been studied and their features have been previously reported [106,107,108,109,110,111]. In particular, studies for C8:0–C18:0 [112,113,114,115,116] may provide powerful identification tools. The published information, supported by newly recorded spectra, is presented and discussed below.

In sFA, the zig-zag hydrocarbon chains assume an overall linear direction, which generally allows for close chain approaching in the bulk phases, and finally, for diverse polymorphic structures, which subtly differ for odd- and even-numbered acids [108,109,115,116]. Fatty acids with chains shorter than that of C9:0 are liquid at room temperature, while C10:0, with a melting point of 31.3 °C, is marginally crystallizable [117]. The latter, along with the higher FA, show pronounced structural infrared features indicative of the crystalline state. Furthermore, even-numbered fatty acids, such as stearic (C18:0), crystallize in temperature-dependent polymorphs A (triclinic) and B, C, and E (monoclinic with orthorhombic sub-cells) [89,108,118]. Structure C is observed at temperatures higher than 35 °C. At room temperature, the metastable monoclinic structure E transforms to the dominating orthorhombic structure B (shown in Figure 1a), where head-to-head acid pairs in trans-planar (or fully extended) chain geometry are formed [89,103,118,119]. This allows the alignment and packing of hydrocarbon chains.

To better study FA infrared spectra, the spectra can be divided into seven regions, each generally having a particular vibrational character. Maxima and assignments are shown in Table 1.

##### The Acidic Hydroxyl Stretching Region

**Figure 2:**
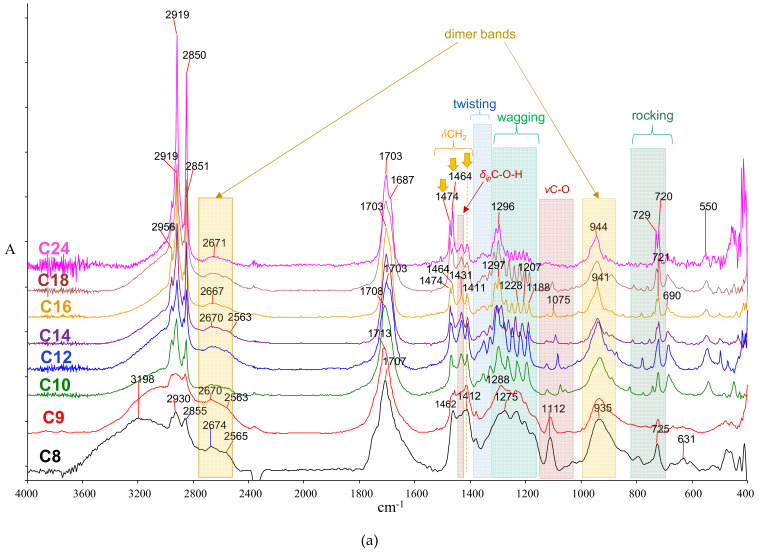

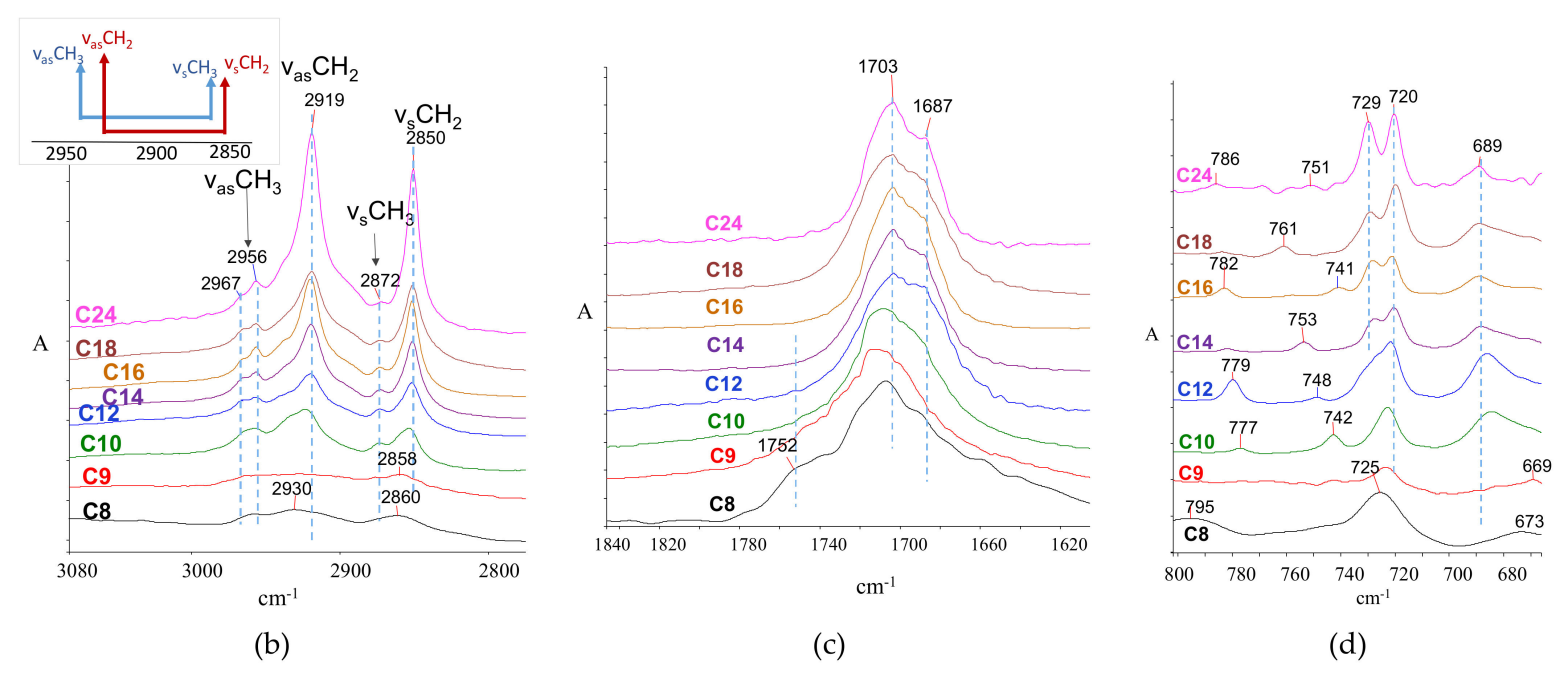
Infrared spectra of fatty acids. (**a**) Full spectra with shaded areas highlighting characteristic features; (**b**) C‒H stretching; (**c**) carbonyl stretching; and (**d**) rocking vibrations. In all spectra: (i) C8, octanoic; (ii) C9, nonanoic; (iii) C10, decanoic; (iv) C12, dodecanoic; (v) C14, tetradecanoic; (vi) C16, hexadecanoic; (vii) C18, octadecanoic; (viii) C24, tetracosanoic. The negative peak at ~2380 cm^−1^ for C8 corresponds to carbon dioxide as a result of improper background subtraction.

The hydroxyl group is involved in the intense hydrogen bonding between molecules of the same type, which controls the structural conformations in the unit cell (Figure 1a). The O‒H stretching region contains a typically broad, intense band, indicative of the acidic OH hydrogen bonding at 3600–2800 cm^−1^ with a vague maximum of around 3050–3000 cm^−1^ (Figure 2a).

An additional broad band at 2750–2500 cm^−1^ is assigned to hydroxyl-carbonyl interactions due to acid dimers [120]. Weak maxima are also observed in this region at ~2670 and 2560 cm^−1^, often labeled as ‘satellite bands,’ and are somewhat unclear as they have been assigned to contributions from combination/summation of *δ*OH and *v*C‒O, Fermi resonance, as well as ionic resonance structures [57,121,122,123]. Nevertheless, they may work as supporting evidence for detecting FAs through infrared spectra of complex mixtures [57].

##### The C‒H Stretching Region

The structural similarities between hydrocarbon chains in sFA and n-alkanes were recognized early and associated with their infrared spectra [107,109], although crucial differences were also pointed out [124]. The C‒H stretch region contains the methyl (CH_3_) and methylene (CH_2_) vibrations, each of these sub-categorized into antisymmetric (*asym*-) and symmetric (*sym*-) vibrations [58,59,108,109,125], the former always observed at higher wavenumbers than the latter (Figure 2b). The maxima depend on the methyl or methylene group proximities to the carbonyl group (the closer to the carboxyl group, the higher the frequency) and the hydrocarbon chain length. Interestingly, the *v*_as_CH_2_ maxima for acids C3 and C4, with only *α*- and *β*- methylene groups (spectra not shown here), appear as high as 2990 and 2973 cm^−1^, respectively [108,126]. Additional CH_2_ groups that build longer chains contribute to the methylene stretching band with a maxima around 2932 cm^−1^ (as shown in the deconvolution analysis in Figure S3a–c). Further addition of CH_2_ groups contributes to the sub-band at 2921–2919 cm^−1^ (Figure S3b,c). For C18:0, the 2919 cm^−1^ peak is the main contributor to the antisymmetric band.

**Figure 3:**
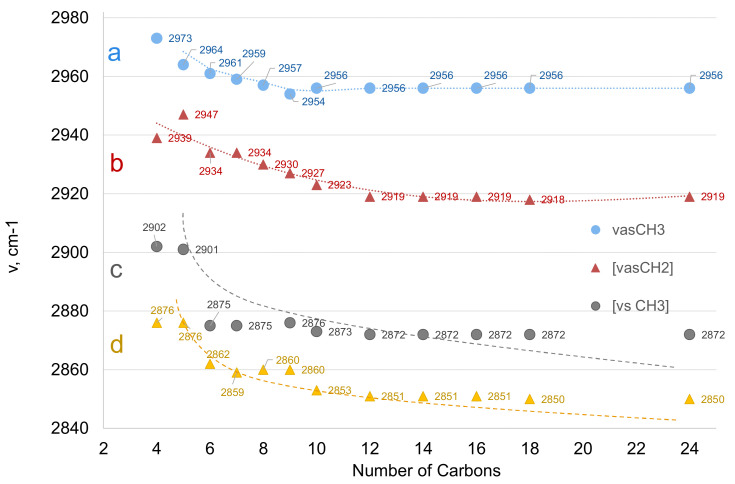
Graph illustrating the trends for (a) *v_as_*CH_3_, (b) *v_as_*CH_2_, (c) *v_s_*CH_3_, and (d) *v_s_*CH_2_ peak maxima with respect to the total number of carbons in sFA. Lower-chain acids, not discussed in the main text, are included to further emphasize the trend.

A trend for *v*_as_CH_3_, *v*_as_CH_2_, *v*_s_CH_3,_ and *v*_s_CH_2_ maxima as a function of the hydrocarbon chain length can be seen in Figure 3. The maxima decrease from short to mid-size (C12) chains, beyond which no significant change is marked (within the spectra acquisition resolution: 4 cm^−1^). Moreover, the intensity ratios of methylene over methyl peaks are related to the number of CH_2_ groups in the chain, thus reflecting the hydrocarbon chain length.

##### The Acidic Carbonyl Stretching Region

The carbonyl group absorption maxima depend on the conformation and packing of the hydrocarbon chains, which are more intense in long chains. The acidic carbonyl stretching vibration maximum (*v*C‒O) is generally observed around 1710–1700 cm^−1^ (Figure 2a); this maximum is considerably downshifted compared to that of isolated molecules (for instance, in the gas phase) [59]. Since the acidic carbonyl group participates in intermolecular hydrogen bonding with the hydroxyl group of a pairing molecule (Figure 1a), the acidic carbonyl downshifts and relatively broadens (Figure 2c) [58,59]. At room temperature, even-numbered acids from C10 and higher are solid and associate efficiently in their crystal; as a result, hydrogen bonding is stronger and results in moderately down-shifted peaks at ~1700 cm^−1^. An additional significant feature is the shoulder at 1687 cm^−1^ observed in C14–C24 (Figure 2c), which can be assigned to a tighter association between dimers.

##### The CH_2_ and CH_3_ Bending Region

The methylene vibration is typically observed at 1465–1430 cm^−1^ for all sFA due to the symmetric in-plane bending (or ‘scissoring’) mode, as shown in Figure 2. A sharp, medium-strong peak around 1470 cm^−1^ is observed. For longer alkyl chain acids, the band is resolved in two sub-bands (~1473 and 1464 cm^−1^) with a contribution of the α-CH_2_ group at ~1411 cm^−1^. On the other hand, the CH_3_ symmetric bend (the ‘umbrella’ vibration) appears at around 1370–1350 cm^−1^, while the antisymmetric appears at 1473 cm^−1^ [56,58,59,127,128]; the relative intensity of these bands generally diminishes with increasing hydrocarbon chain length. Figure S1 zooms in the 1570–1000 cm^−1^ region, showing bands more clearly for the crystallizable FAs.

##### The CH_2_ Twisting and Wagging Region

This region is characteristic of crystallizable compounds and materials containing long saturated hydrocarbon chains; as a typical feature, band splitting in the form of band progressions appears [59] (shown in Figure 2a and, in more detail, in Figure S1). Early systematic studies that started in the 1950s, based on the coupled oscillator model of the zigzag-shaped hydrocarbon chain [107,124,129,130], offered a better understanding of this phenomenon. In the crystalline state, the twisting vibration of sFA is split throughout 1368–1329 cm^−1^ which better resolves upon chain increase; double-split CH_2_ twisting peaks are observed in C14:0-C18:0, while four-split are observed in C24:0.

A similar phenomenon is even more pronounced for the methylene wagging vibrations, where extensive splitting in the form of progressions at 1320–1160 cm^−1^ are observed [107,108,109,110,131]. The multiplicity of progressions (which can be termed as *w*_1_, *w*_2_, etc.) increases with increasing hydrocarbon chain length (seen in detail in Supplementary Materials Figure S1), showing a multiplet of bands, the number *w* of which appears to follow the rule $w=\frac{n}{2}-1$, where *n* is the total number of carbons in a fatty acid. Moreover, the distancing between consecutive *w* bands diminishes with increasing hydrocarbon chain, approx. 34 (for C10), 29 (C12), 24 (C16), 18 (C18), and 13 cm^−1^ (C24). The progressions may be stronger in well-crystallized samples, for instance, when the crystalline state is slowly formed upon the annealing of a fatty acid melt [108,109,132,133]; this is demonstrated in Figure S3 for stearic acid (C18:0), where a recrystallized C18:0 sample after a heating-annealing cycle is compared with the one that was recorded as purchased. Generally, on a practical level, when these bands are distinguishable, the carbon number of the fatty acid can be estimated.

##### Hydroxyl C‒O Stretch and C‒O‒H Bending Region

The carbon-oxygen bond of the acidic hydroxyl stretches (*v*C‒O) at around 1100 cm^−1^ and appears as a medium peak (Figure 2a); for liquid fatty acids, the band is relatively broad at 1100–1110 cm^−1^, while for solid acids, it is split at 1124 cm^−1^ (weaker) and 1075–1122 cm^−1^ (stronger). The latter upshifts upon moving from C10:0 to C18:0, while in C24:0 this peak almost disappears (Figure S1).

The C‒O‒H in-plane bending vibration (*δ_ip_* C‒O‒H) is observed as a medium, relatively broad peak at ~1431 cm^−1^ [59]. Although it is often partly overlapped in unknown, complex samples, its diagnostic value can be significant. Likewise, the out-of-plane bending vibration (*δ*_oop_ C‒O‒H), which typically appears as a weak peak at around 690 cm^−1^ and is also responsible for a medium-strong broad peak at 943–935 cm^−1^ [56,59] due to dimer formation, is also of diagnostic value (Figure 2a).

##### The CH2 Rocking Region

This band may span in the 780–700 cm^−1^ range [124]. In-phase CH_2_ rocking vibration most prominently appears at 725–710 cm^−1^ (Figure 2d). In the crystalline state (and similarly with hydrocarbons), the band splits into a doublet with *Δv* 7–10 cm^−1^, due to the anisotropic coupling of similar vibrations in adjacent CH_2_ groups [109,134]. As shown in Figure 2d, splitting intensifies for long hydrocarbon chains (C14:0, C16:0, C18:0, and even more for C22:0).

##### Infrared Maxima and Crystallization

Most spectral characteristics discussed above depend on the state of fatty acids. Efficient crystal packing results in the downshifting of acidic carbonyl and the formation of CH_2_ structural features (twisting, wagging progressions, and splitting of bending and rocking vibrations). On the other hand, non-crystallizable sFA, such as C8:0 and C9:0 (melting points 16.5 °C and 12.4 °C, respectively) show a relatively uniform broad band at 1380–1180 cm^−1^ with poorly resolved shoulders (Figure 2a); the lack of multiplicities reflects their non-crystalline nature at room temperature. A marginal case is capric acid (C10, melting point 34 °C), where crystallization at room temperature is not always efficient and although the wagging progressions are evident, the carbonyl peak appears upshifted (1708 cm^−1^), and the rocking vibration is not split (Figure 2a,c).

The rocking band is significant and of diagnostic value regarding the chain length and effectiveness of crystallization. While the twisting and wagging progressions (discussed above) appear for any solid-state FA (i.e., for C10 and higher), the rocking band splits for chains longer than C14, as shown in Figure 2a,d. Furthermore, effective crystallization may affect the shape of the split band (i.e., even and deeper-split sub-bands in well-crystallized samples). The crystallization effect is shown in Figure S3 by comparing the CH_2_ rocking band of C18:0 before and after the annealing-recrystallization cycle. In conditions favoring even more efficient packing (such as freezing in 10K [124]), the rocking vibration is even more split where coupling with the twisting vibration is evident as a progression ranging from 1065 to 720 cm^−1^.

#### 2.2.2. Fatty Monoacid Metal Salts

Fatty acids can be transformed into FAMS in the presence of metals [135,136] and reactive metallic compounds such as oxides, hydroxides, and salts [62,71,137,138,139]. In paintings, many of these metallic compounds exist in inorganic pigments and have been found to cause soap formation, particularly of zinc and lead, a phenomenon that has received considerable attention from researchers due to the detrimental effects in works of art [5,77,87,140,141,142]. Their presence in archaeological samples and other cultural heritage objects has also been documented [8,9]. In particular, calcium soaps are considered to be common products through FA adsorption to calcium carbonate in the geological environment and ceramics [27,143,144], since their formation is favored due to their very low solubilities in water (*K*_sp_ of the order of 10^−17^ mol^3^/L^3^). Other metal soaps can also be expected, such as copper, aluminum, cobalt, and iron [84,139,145]. This mineralization process, which can be followed by inspecting infrared spectra of samples, may prolong the preservation of the lipid fraction in the burial or other environments due to low solubility. Moreover, the above may lead to a severe underestimation of lipid yields during routine chromatographic analysis involving lipid extraction with organic solvents [27]. In the light of the above, chromatographic analysis methodologies measuring metal soaps as added components in lipid analysis of cultural heritage samples have recently been considered [146]. Finally, contamination by cleaning agents, including the sodium soaps (typically, stearates), may not be excluded. For all the above reasons, identifying FAMS in the sample prior to any chemical processing offers significant help in assessing the overall lipid load in a sample and further assessing its prehistory. To this end, the infrared spectra of calcium, zinc, and sodium fatty acid salts are presented, and their characteristic features are pointed out.

The infrared spectra of FAMS have been studied to some extent [78,87,133,138,147]. In support of the current discussion, selected sodium (representative of late-historical cleaning agents and modern additives in routinely used materials), calcium (typically expected in interaction products of FA with the calcareous environment) and zinc soaps (a usual case of metal soaps in oil paintings) were prepared in the laboratory (see Materials and Techniques). Their infrared spectra were recorded.

**Figure 4:**
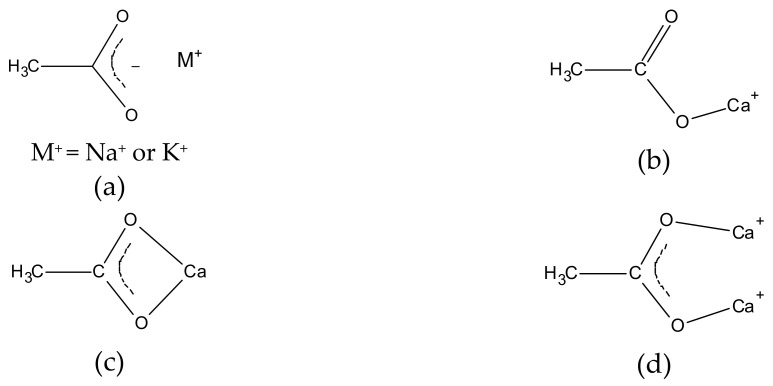
Chemical structures of metal carboxylates. (**a**) Ionic form of monovalent metal ions; (**b**) unidentate coordination; (**c**) chelating bidentate coordination; and (**d**) bridging bidentate coordination to calcium ions. Structures adapted from [148,149,152].

**Figure 5:**
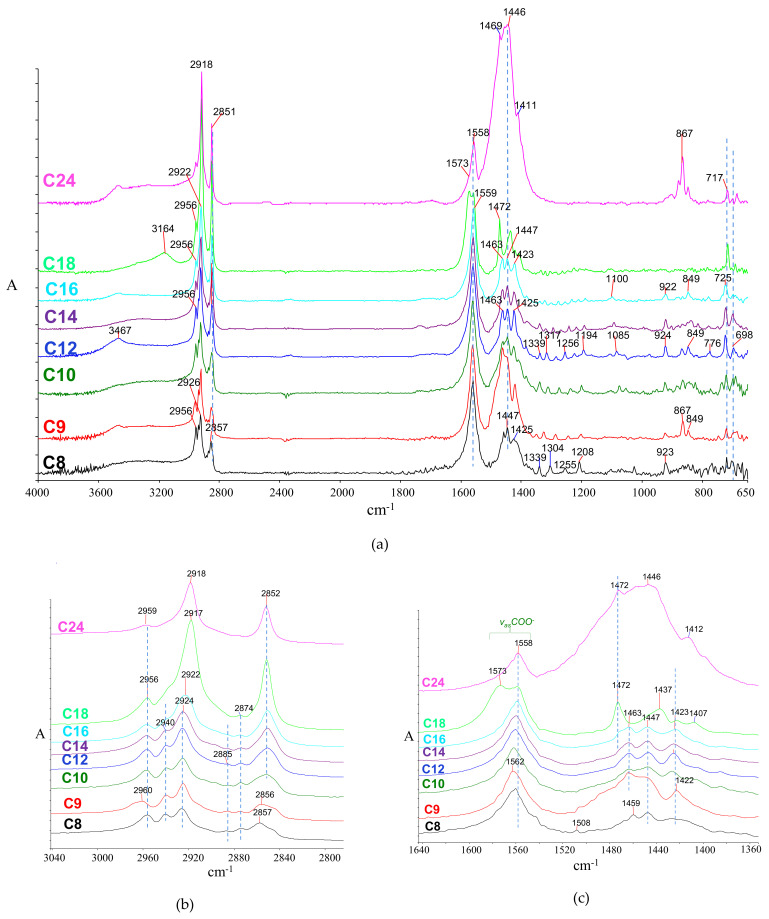
Infrared spectra of fatty acid sodium salts. (**a**) Full spectrum; (**b**) C‒H stretching region; and (**c**) carboxylate region. In all spectra: C8, sodium octanoate; C9 sodium nonanoate; C10, sodium decanoate; C12, sodium dodecanoate; C14, sodium tetradecanoate; C16, sodium hexadecanoate; C18, sodium octadecanoate; C24, sodium tetracosanoate; C9 and C24 are contaminated with sodium carbonate (intense peaks at ~1450 and 867 cm^−1^). The negative peaks at ~2380 cm^−1^ correspond to carbon dioxide as a result of improper background subtraction.

Although soap spectra generally share similar features, diagnostically significant differences can be observed regarding the carboxylate and some of the hydrocarbon chain vibrations. Moreover, in the case of the bivalent ions Ca and Zn, hydrated salts were produced, as evidenced by the intense, broad, crystalline water peaks (at ~3440 and ~1626 cm^−1^ for the calcium salt, and ~3565–3580 and 1620–1600 cm^−1^ for the zinc salt). Spectra are shown in Figure 3 (sodium), Figure 4 (calcium), and Figure 5 (zinc), while peaks with their assignments are listed in Table 2.

##### The Carboxylate Stretching

The carboxylate bands typically exist in pairs corresponding to the antisymmetric (*v_as_*COO^−^) and symmetric (*v_s_*COO^−^) carboxylate vibrations at 1575–1530 and 1460–1400 cm^−1^, respectively.

The coordination geometry plays a significant role in the band maxima [147,148,149]. Moreover, the separation between the antisymmetric and symmetric band, often called the *Δ* (‘delta’) value, depends on the coordination symmetry; the lower the symmetry, the higher the *Δ*. More specifically, carboxylates may assume various symmetry levels based on whether they exist in a pure ionic form (or a salt) or metal-coordinated forms such as the unidentate, the chelate bidentate, and the bridging bidentate [148,149,150,151] (Figure 4). In the lower-symmetry unidentate complexes (Figure 4b), the antisymmetric maximum is expected to appear close to that of the corresponding carboxylic acid. The more symmetric ionic and chelating bidentate structures (Figure 4a,c, respectively) appear at roughly similar frequencies, while the bridging bidentate is even lower [149]. The maxima are very sensitive to the metal type (i.e., ion mass, electronegativity, and effective charge [87]) but generally insensitive to alkyl chain length, as shown in Figure S4.

The sodium soaps can be correlated with the ionic structure (Figure 4a), showing carboxylate maxima at ~1560 cm^−1^ and 1425 cm^−1^ for *v*_as_COO^−^ and *v*_s_COO^−^, respectively [149]. Specifically, for sodium stearate (C18Na), the antisymmetric band appears as a doublet at 1573 and 1559 cm^−1^ (Figure 5c), possibly due to two different sub-cell arrangements [153]. Interestingly, deviations of the above are observed in cases where sodium salts co-crystallize with their corresponding FA. A unified acid-soap carbonyl stretch is observed in above, at ~1740 cm^−1^, attributed to a hydrogen bonding decrease combined with changes in acid-soap conformations [153].

**Figure 6:**
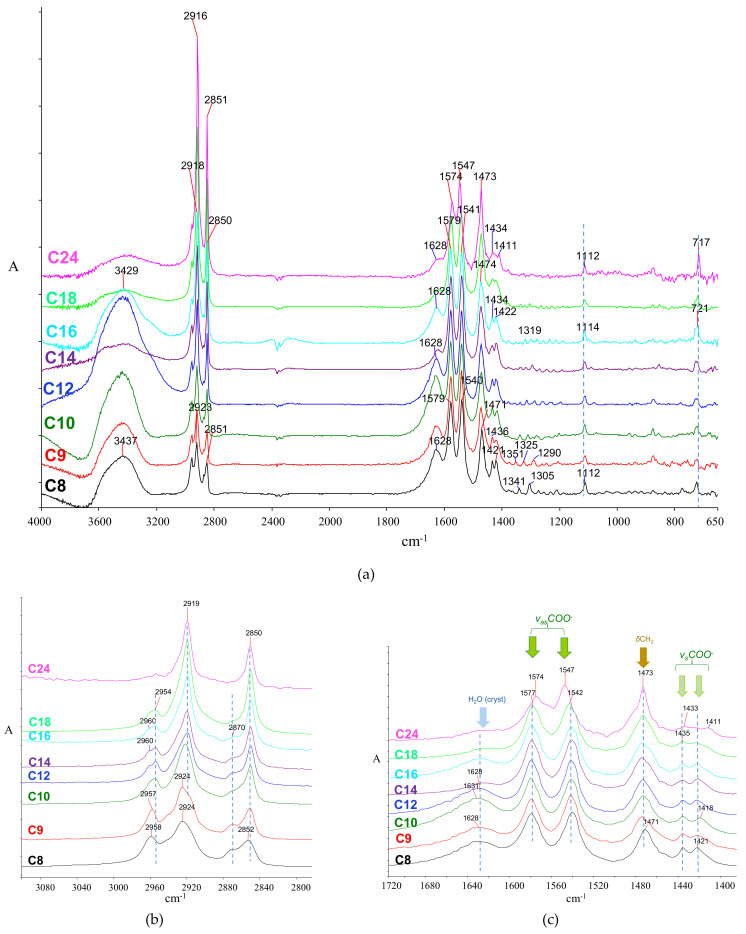
Infrared spectra of fatty acid calcium salts. (**a**) Full spectrum; (**b**) C‒H stretching region; and (**c**) carboxylate region. In all spectra: C8, calcium octanoate; C9 calcium nonanoate; C10, calcium decanoate; C12, calcium dodecanoate; C14, calcium tetradecanoate; C16, calcium hexadecanoate; C18, calcium octadecanoate; C24, calcium tetracosanoate. Contaminated with small amounts of calcium carbonate (peaks at ~1435 and 874 cm^−1^). The negative peaks at ~2380 cm^−1^ correspond to carbon dioxide as a result of improper background subtraction.

For the calcium soaps, double-splitting is observed for all FAMS at 1580 and 1542 cm^−1^ (*v*_as_COO^−^) and 1435 and 1419 cm^−1^ (*v*_s_COO^−^), indicating two different denticities in the coordination structure (Figure 6). Splitting of this type has been reported for precipitated calcium soap molecular units in bulk acquiring three-dimensional molecular geometry [84,154]. On the other hand, for two-dimensional calcium soap layers, such as those formed through the interaction of fatty acids with calcium-containing surfaces, the doublet becomes a singlet (1550 cm^−1^ for the antisymmetric band) [155]. Since the calcium soaps as reaction products of free fatty acids with calcium carbonate are generally expected in specific archaeological contexts [27,143,144], the above maxima are generally expected and have been in some instances observed [8,9].

**Figure 7:**
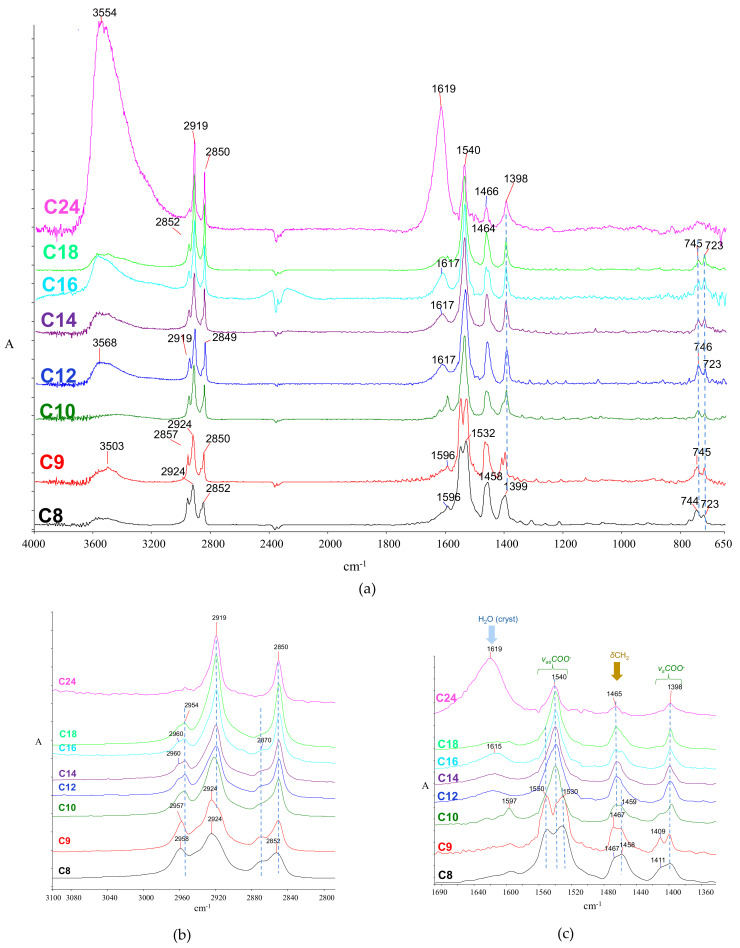
Infrared spectra of fatty acid zinc salts. (**a**) Full spectrum; (**b**) C‒H stretching region; and (**c**) carboxylate region. In all spectra: C8, zinc octanoate; C9 zinc nonanoate; C10, zinc decanoate; C12, zinc dodecanoate; C14, zinc tetradecanoate; C16, zinc hexadecanoate; C18, zinc octadecanoate; C24, zinc tetracosanoate. The negative peaks at ~2380 cm^−1^ correspond to carbon dioxide as a result of improper background subtraction.

The zinc salts (Figure 7) in most cases show single, relatively broadened carboxylate bands at 1540 (*v*_as_COO^−^) and 1398 cm^−1^ (*v*_s_COO^−^). However, in the case of the shorter-chain fatty acid salts (C8_2_Zn and C10_2_Zn), splitting is observed at 1550 and 1532 cm^−1^ (*v*_as_COO^−^), and 1410 and 1399 cm^−1^ (*v*_s_COO^−^) [72,73,155].

As a general remark, the splitting in both *asym*- and *sym*- carboxylate bands of the divalent metal salts into doublets has been previously observed and attributed to bidentate and unidentate salt types (Figure 4) [154,156]; the splitting can be as large as ~40 cm^−1^ for the calcium salts, while it is significantly smaller for the zinc salts. The trends in antisymmetric and symmetric carboxylate maxima for various sodium, calcium, and zinc soaps are shown in Figure S4. A significant feature of the carboxylate bands is the separation Δ*ν* between the antisymmetric and symmetric peaks, considered to depend on factors such as the type of metal, the ligand, and the coordination type and denticity [157,158], the packing of chains [158,159,160,161], as well as the molecular environment (solvent, or other materials in the molecular environment) [73,160]. As shown in Figure S4 (values drawn from spectra in Figure 4, Figure 5, and Figure 7), the Δ*ν* value in the lower-symmetry sodium soaps is generally expected to be ~137 cm^−1^, while for the higher-symmetry calcium and zinc, this is expected to be higher at 142–144 cm^−1^ [158].

The calcium and zinc salts show similar Δ*v* values at 143–144 cm^−1^ (with the exception of the C24 salt), suggesting higher-symmetry bidentate coordination. The differences in separation values between the sodium and the divalent salts are marginal as they are comparable with the wavenumber uncertainty limit (4 cm^−1^, see Materials and Techniques). Our data shows a deviation for the C8Zn and C9Zn salts, showing Δ*ν* values at 131 cm^−1^.

As a general remark, the carboxylate bands can be significantly broadened and shifted so that Δ*v* decreases when soaps are formed in an amorphous molecular vicinity (as is the case of metal soaps formed in paintings with the glassy molecular environment of the binding medium) [6,73,160].

##### The C‒H Vibrations

As a general remark, the CH_2_ antisymmetric stretching band in FAMS shows fewer components and lower variability in their maxima than those in FAs. Spectra are shown in Figure 3b, Figure 4b, and Figure 5b, for the three metal salts, respectively. For the carbons closer to the carboxylate group, maxima appear at higher frequencies (2945–2935 cm^−1^), while for the more distanced ones, these are downshifted at approximately 2920 cm^−1^. Notably, for the sodium salts of fatty acids with chains shorter than C14, the peaks are resolved to ~2957, 2940, and 2920 cm^−1^. The *sym*- CH_2_ stretching typically appears at 2857–2850 cm^−1^, and the CH_3_ stretching appears at 2956 (*asym*-) and 2874 cm^−1^ (*sym*-).

The methylene bending vibrations were found at 1460–1470 cm^−1^ (Na salts), 1468–1473 cm^−1^ (Ca salts), and 1456–1467 cm^−1^ (Zn salts), respectively, while the methyl bending is either very weak or not observed at all. This region is shown in Figure 3c, Figure 4c, and Figure 5c. The twisting vibrations are observed at 1400–1260 cm^−1^ as progressions of four or five peaks, which are quite intense for Ca and Zn salts. The wagging vibrations are also observed as progressions, generally at 1270–1180 cm^−1^, similar to those of the corresponding FA, although relatively weaker and at numbers that follow the n/1 pattern (where n is the number of total carbon atoms). The weak bands at 870–860 cm^−1^ are assigned to the coupling between *δ*CH_3_ and *v*C-C [84]. Finally, the splitting of rocking vibrations in the form of doublets at 745–723 cm^−1^ is observed only for zinc salts (Figure 7a). In most other cases, this is elusive, reflecting a misalignment of the hydrocarbon chains, possibly induced by the big difference between the ionic group and hydrocarbon chain polarities.

Based on the recorded spectra, the above results refer to the pure materials, and therefore ‘ideal’ situations in the absence of organic additives, inorganic materials, etc.; spectra of soaps present in ‘real’ samples may differ in their subtle features such as progressions and splitting. Metal soaps on surfaces may acquire liquid crystal character [138]; in the case of a divalent metal (calcium, zinc), the hydrocarbon chains arrange on both sides of the metal ion plane. When this arrangement is unperturbed (for instance, in pure salts), alkyl chains longer than C12 align well to show the progressions and splittings, as described above. However, the non-polar liquid crystal layer may occasionally accommodate foreign compounds of small MW (solvents, degradation products, etc.) that may disrupt the ordered chain alignment and, therefore, diminish the intensity of these features [138,145,161]. In these cases, the appearance of progressions and split peaks can be considered as a measure of phase purity. Nevertheless, more research is needed to investigate the crystallization phenomena of metal soaps in various environments.

### 2.3. Infrared Spectra of Selected Diacids and Their Metal Salts

#### 2.3.1. Azelaic and Suberic Acids

**Figure 8:**
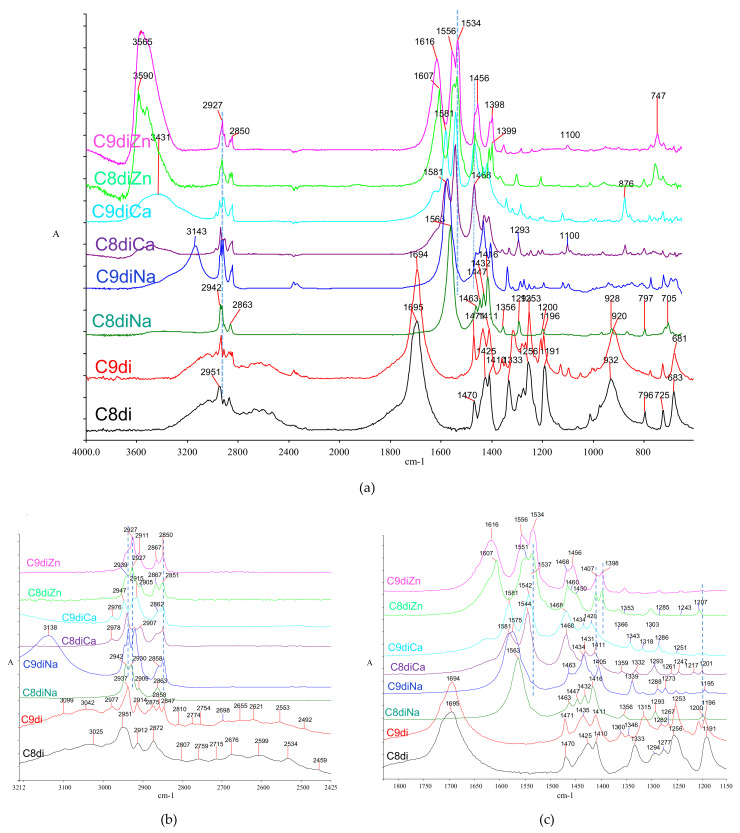
Infrared spectra of selected dicarboxylic acids and their sodium, calcium, and zinc salts. (**a**) Full spectrum; (**b**) C‒H stretching region; and (**c**) 1800–1150 region. In all spectra: C8di, octanedioic acid (suberic acid); C9di, nonanedioic acid (azelaic acid); C8diNa, C8diCa, and C8diZn, sodium, calcium, and zinc octanedioates (suberates), respectively; C9diNa, C9diCa, and C9diZn, sodium, calcium, and zinc nonanedioates (azelates), respectively.

During the last 50 years, research has shown that diacids, especially nonanedioic and octanedioic (azelaic, C9di, and suberic, C8di, respectively) acids, are standard oxidation products of polyunsaturated fatty acids [75,84,162]. They are commonly detected in organic remains of archaeological objects containing unsaturated oils [1,2,10,163,164,165], and in oil-based paintings [76,166,167,168,169]. Specific features in diacid spectra (shown in Figure 8, maxima and assignments in Table 1) can be helpful in their identification and distinction from monoacids.

The acidic carbonyl absorption for diacids is observed at ~1695 cm^−1^, downshifted compared to monoacids due to more efficient hydrogen bonding between carboxyl groups. According to their structural characteristics, carboxyl groups are associated with intense intermolecular hydrogen bonding, resulting in monoclinic crystals. As seen in Figure 1b,c, the structures differ depending on odd- and even-numbered chains [91,170,171,172,173].

Similar to monoacids, the hydroxyl stretching is typically observed as a broad band, spanning 3200–2800 cm^−1^. On the other hand, and in contrast to monoacids, the hydroxyl dimer band is structured, showing at least seven characteristic satellite bands spanning the 2810–2459 cm^−1^ region (Figure 8b).

Besides the expected absence of methyl bands, which can be seen as a diagnostic feature in spectra, the methylene stretching region is characteristically more complex than those of monoacids with split antisymmetric and symmetric bands. In the case of azelaic acid, this is more intense with both the *v_as_*CH_2_ and *v_s_*CH_2_ bands triply resolved at 2977–2914 cm^−1^ and 2875–2847 cm^−1^, respectively (Figure 8b). The difference in the CH_2_ stretching bands between the two acids can be attributed to a different crystal geometry allowing polymorphism in their monoclinic structures based on parallel and vertical orientations among the carboxyl group planes, resulting in different crystal packing between even- and odd-numbered diacids [90,91,115,172,173,174,175,176,177,178,179].

The methylene bending vibrations are observed as doubly split bands at ~1470 and 1410 cm^−1^ (Figure 8c). The twisting vibrations are triply split (1360, 1346, and 1315 cm^−1^) in azelaic acid, while they show a uniform peak in suberic acid (1333 cm^−1^). The wagging vibrations are triply split in both acids at 1290–1250 cm^−1^. The rocking vibration appears as two medium-weak peaks at ~796 and 725 cm^−1^ for both acids with no evident splitting.

The hydroxyl bending (*δ*C‒O‒H) appears as an obscure band at 1425 and 1435 cm^−1^, for C8di and C9di, respectively [115] (Figure 8c). The acidic carbonyl C‒O stretch is a very weak doublet at 1105 and 1098 cm^−1^ for azelaic, while it is not observed for suberic acid (Figure 8a). Finally, the out-of-plane C‒O‒H bending is shown at 681 cm^−1^ for both acids, while its dimer counterpart appears as a broad band at 932 (suberic) and 920 cm^−1^ (azelaic).

#### 2.3.2. Diacid Metal Salts

In the case of diacid salts, most research has been done in the art paintings context. However, the literature for their systematic infrared spectra is limited [84,180], despite the fact that the corresponding fatty acids are widespread in oil paintings and specific archaeological samples, and the formation of their salts through ion exchange is more than expected. As a result of their difunctional character, diacid metal salts may associate with divalent metal ions with both their ionic ends; this way, metal-coordinated ionic networks can be formed with an ionomer character [6,75,145], which through their diminished mobility may act as a stabilizing means for the medium. This structural phenomenon has been proposed as a ‘self-repair’ mechanism by diacid salts which mitigates the detrimental effect caused by soap formation in paintings [84,145]. Although diacids have been investigated in lipid-containing archaeological samples [164], diacid salt formation has not been reported, and therefore, no data exist for similar phenomena in the various archaeological environments.

Similarly, with the previous cases, suberate (C8 dicarboxylate, or C8di), and azelate (C9 dicarboxylate, or C9di) salts with sodium, calcium, and zinc were also synthesized in the laboratory (see Materials and Methods). As seen in the spectra of Figure 8, the main infrared features of suberates and azelates (listed in Table 2) are similar to those of monoacids, although with subtle, but important differences. Specifically, the zinc salts appear in their hydrated form, with crystalline water observed at 3560–3520 cm^−1^ and 1607, and 1616 for C8di and C9di, respectively (Figure 8a). The carboxylate bands are intense and characteristic, appearing as an *asym*-/*sym*- pair (Figure 8c). For the sodium salts, a pair of single carboxylate peaks are observed at 1563–1575 (*asym*-) and 1416–1405 (*sym*-) (Figure 8c). On the other hand, each vibration is doubly split at ~1575–1544 (*asym*-) and 1431–1410 (*sym*-) for calcium and ~1551–1534 (*asym*-) and 1412–1400 (*sym*-) for zinc. By analogy to monoacid salts, the splitting can be attributed to differences in coordination geometry. The splitting value (*Δv*_split_) is 40 cm^−1^ for the calcium salts, while it is significantly smaller, ~18 cm^−1^, for the zinc salts.

Most methylene bands in diacid salts are characteristically structured, possibly as a result of interactions in the crystalline phase (Figure S1b,c). The antisymmetric CH_2_ stretching band is split into four components for the calcium salts at 2978–2907 cm^−1^, four components for sodium, and three for zinc salts at 2940–2905 cm^−1^ (see Figure 8b). The CH_2_ bending band (scissoring) is also structured at 1463–1405 cm^−1^, with the suberate band being more extended than the azelate. The methylene twisting is observed as a single peak at ~1360 cm^−1^ for most salts, while wagging appears as a progression at 1318–1195 cm^−1^, significantly more extended for the azelates. Finally, the CH_2_ rocking is observed at 725–705 cm^−1^ for the sodium and calcium diacid salts and 747, 723 cm^−1^ for the zinc salts.

## 3. Materials and Methods

### 3.1. Materials

Fatty acids were purchased from Sigma-Aldrich (Kenilworth, NJ, USA): octanoic, or caprylic (CH_3_(CH_2_)_6_COOH or C8:0, ≥99%), nonanoic, or pelargonic (CH_3_(CH_2_)_7_COOH or C9:0, ≥99%), decanoic, or capric (CH_3_(CH_2_)_8_COOH or C10:0, >98.0%), dodecanoic, or lauric (CH_3_(CH_2_)_10_COOH or C12:0, ≥99%), tetradecanoic, or myristic (CH_3_(CH_2_)_12_COOH or C14:0, ≥99%), hexadecanoic, or palmitic (CH_3_(CH_2_)_14_COOH or C16:0, ≥99%), octadecanlic, or stearic (CH_3_(CH_2_)_16_COOH or C18:0, ≥98.5%), tetracosanoic, or lignoceric (CH_3_(CH_2_)_22_COOH or C24:0, ≥99%), octanedioic, or suberic (C_6_H_12_(COOH)_2_ or C8di, 98%), and nonanedioic, or azelaic (C_7_H_14_(COOH)_2_ or C9di, 98%).

Moreover, additional material and reagents were acquired as follows: sodium hydroxide (Sigma-Aldrich >88%), water (Honeywell, Charlotte, NC, USA, HPLC grade), calcium chloride (anhydrous, Sigma-Aldrich, ≥93%), zinc chloride (Sigma-Aldrich, ≥98%), ethanol (Merck, Kenilworth, NJ, USA, 99.5%), chloroform (Sigma-Aldrich, anhydrous, ≥99%), xylene (mixture of isomers, ≥98.5%), and acetone (Honeywell, ≥99.5%).

### 3.2. Synthesis of Fatty Acid Metal Salts

Sodium salts of fatty acids C8:0–C24:0 were prepared by adding 0.1 mmol of the corresponding acids to 1 mL of sodium hydroxide 0.1 M solutions in ethanol (Honeywell); for the diacid (C8di and C9di) sodium salts, two equivalents of the base was added. The solutions were initially warmed up to 80 °C on a heating plate, followed by sonication for 15 min. When precipitation was complete, the products were investigated with infrared spectroscopy, where the full conversion to the sodium salt was confirmed for all cases.

The calcium and zinc salts of fatty acids C8:0–C24:0 were prepared by substitution upon mixing aqueous solutions of the corresponding sodium salts (prepared in the previous step and pre-heated on a plate for full solubilization) with aqueous solutions of calcium chloride (Merck, Kenilworth, NJ, USA) and zinc chloride (Sigma-Aldrich, Kenilworth, NJ, USA). The mixtures were sonicated until full conversion was evident through infrared spectroscopy. Infrared spectra of obtained salts (shown in this paper) were in agreement with the literature [71,78,82,83,84,85].

### 3.3. Fourier Transform Infrared Spectroscopy

All samples were in powder form and analyzed according to the following procedure: each sample was mixed with KBr, pulverized in a pestle and mortar, and pressed in a 13 mm disc using a hydraulic press. Infrared spectra of the KBr discs were recorded in transmission mode using a Perkin Elmer Spectrum GX 1 FTIR spectrometer equipped with a DTGS detector at 4000–650 cm^−1^, 32 scans, and 4 cm^−1^ resolution. Spectra of acids are shown normalized on the carbonyl peak. Spectra of salts are shown normalized on the highest carboxylate peak. Deconvolution of the C‒H region (3000–2800 cm^−1^) was done using the Peak Fitting application of GRAMS/AI (Thermo) software, using a mixed Gaussian (50%) and Lorentzian (50%) function, at low sensitivity. In all cases, the standard error was lower than 0.00647, while R^2^ was better than 0.9987.

## 4. Conclusions

The investigation of fatty acids and their metal salts as degradation products of fatty substances in oil paintings and particular archaeological objects can be of high importance. This work explores the usability of infrared spectroscopy for characterizing these compounds in related complex samples. The spectra of some typical fatty monoacids, diacids, and their sodium, calcium, and zinc salts are investigated, and their features were examined in terms of their diagnostic usability.

Intermolecular interactions are fundamental for the acidic carbonyl maxima, which downshift from 1713 (for C9 monoacid) to 1703 cm^−1^ (C14–C24); these are further downshifted to 1694–1695 cm^−1^ in diacids. Crystallization and effective molecular associations in the higher members are key factors for this phenomenon, and in some cases, they are inhibited due to unfavorable molecular environments; acidic carbonyl maxima are relatively high, at ~1710 cm^−1^. The same phenomenon also causes broadening of the dimer band at 945–920 cm^−1^.

In salts, carboxylate absorptions exist in antisymmetric-symmetric pairs (at 1590–1530 and 1436–1398 cm^−1^, respectively), often existing in doublets, with separations (Δ*v*) depending on the metal. The distinctly higher frequency (181–1579 cm^−1^ of the calcium salts is helpful for their identification. In these maxima, chain lengths have little or no effect. On the other hand, inefficient crystallization may affect the band envelopes and the separation of both bands. The hydroxyl stretch spans the 3200–2800 cm^−1^ region of acids with a maximum of around 3000 cm^−1^. Dimer formation is a typical feature in carboxylic acids, characteristically appearing as a broad maximum involving the stretching vibrations at 2800–2400 cm^−1^ (in diacids, it appears as a structured band) and as a relatively broad band involving the bending vibrations at ~940 cm^−1^.

The various C‒H vibrations significantly depend on monoacid chain lengths and intermolecular interactions among them. In crystallizable samples, splitting in stretching, bending, and rocking vibrations and the characteristic progressions in twisting and wagging for the longer-alkyl chain acids are often a diagnostic asset. Diacids also show splittings but no progressions. Progressions are also expected in metal monoacid soaps, although significantly weaker and more elusive, and therefore, their diagnostic capability is limited.

The rich spectroscopic features of fatty acids and their salts significantly benefit their diagnostic use. A close inspection of an infrared spectrum recorded from complex samples containing fatty acids, or their metal salts, can provide direct evidence for their condition.

## Figures and Tables

**Table 1:** Main infrared peaks and assignments of saturated fatty carboxylic monoacids and diacids.

Peak Maxima, Wavenumbers (cm^−1^) ^1^			Assignment	Notes
Monoacids	Octanedioic (Suberic) Acid	Nonanedioic (Azelaic) Acid		
3600–2800 br	3037	3045	*v*OH	Typically, very broad with a vague maximum.
2960–2956 m-w	n.a.	n.a.	*v*_as_CH_3_	Variable, according to the number of carbons.
2934–2919 s	2991, **2951**, 2941, 2911	2977, **2937**, 2915	*v*_as_CH_2_	Variable, according to the number of carbons; higher-frequency maxima correspond to a lower distance from the COOH group.
2875–2872 w, s	n.a.	n.a.	*v*_s_CH_3_	Variable, according to the number of carbons.
2851 m-s	2872, 2855	2875, 2858, 2847	*v*_s_CH_2_	Variable, according to the number of carbons.
2670, 2565 br	2759, 2676, 2599, 2534	2774, 2698, 2621, 2553	*v*O‒H•••O=C (dimer stretching band)	Structured with weak shoulders; more extended in diacids.
1703 vs	1695	1694	*v*C=O, acidic	
1472, 1464, 1411 m	1470, 1410	1471, 1411	*δ*CH_2_ scissoring	Split into three components (two in diacids); the 1472 and 1464 cm^−1^ components are better resolved for monoacids C16-C24.
1457–1450 w	n.a.	n.a.	*δ*_as_CH_3_	Contribution is lower for higher carbon-number chains.
1431 m-w	1426	1436	*δ*_ip_COH	Relatively broad; often missed due to overlaps.
1372 m-w			*δ*_s_CH_3_ (‘umbrella’ vibration)	
1356–1347 m-w	1334	1360, 1346	*τ*CH2	Splitting in progressions for acids in their crystalline state (in room temperature, higher than C10).
1318–1185 m-w	1360, 1346	1317, 1282, 1268, 1254, 1208, 1196	*w*CH2	Splitting in extended progressions for acids in their crystalline state (in room temperature, higher than C10).
1112–1075 m-w	n.o.	1105, 1098	*v*C‒OH	Up-shifted for longer hydrocarbon chains. Weak, or not observed for diacids.
943–935 m, br	932	920	*δ*_oop_C‒O‒H•••O=C (bending dimer band)	
795–741, 725–710 w	796, 725	776, 726	*ρ*CH2	Stronger in long hydrocarbon chains; the 725 cm^−1^ peak is doubly split in crystalline monoacids.
690	683	681	*δ*_oop_C‒O‒H	

^1^ in bold: the stronger peaks in the case of multiplets. *v*: stretching vibration; vs. symmetric stretching; *v_as_* antisymmetric stretching; *δ*: bending vibration; *δ_s_* symmetric bending; *δ_as_* antisymmetric bending; *δ*_ip_: in-plane bending; *δ*_oop_: out-of-plane bending; *ρ*: rocking vibration; *τ*: twisting vibration; *w*: wagging vibration.

**Table 2:** Main infrared peaks and assignments of monoacid and diacid metal salts.

Peak Maxima, Wavenumbers (cm^−1^)			Assignment	Notes
Monoacid Metal Soaps	Octanedioate (Suberate) Metal Soaps	Nonanedioate (Azelate) Metal Soaps		
3600–3300	3590, 3521 Zn	3565, 3521 Zn	*v*O‒H (cryst. water)	In Ca and higher Zn monoacid salts. Only in zinc diacid salts.
2956	n.a.	n.a.	*v*_as_CH_3_	
2940(sh), 2926–2916	2942, 2930, 2909 Na 2978, 2939, 2921, 2907 Ca 2939, 2927, 2905 Zn	2943, 2936, 2921, 2907 Na 2976, 2947, 2921, 2915 Ca 2939, 2927, 291 Zn	*v*_as_CH_2_	Downshifted (3–5 cm^−1^) in monoacid salts, for >C16 (Ca, Na) and >C14 (Zn). Structured in diacid salts.
2874–2870	n.a.	n.a.	*v_s_*CH_3_	
2856–2850 Na salts 2851 Ca and Zn salts	2863 Na 2860, 2850 Ca 2867, 2851 Zn	2858, 2848 Na 2862, 2849 Ca 2867, 2850 Zn	*v*_s_CH_2_	Downshifted (~5 cm^−1^) in monoacid Na salts, for >C9. Split into two components in diacid salts
1628 (br) Ca salt 1617–1619 Zn salts C12-C24	1607 Zn	1616 Zn	*δ*H-O‒H (cryst. water)	In Ca and higher Zn monoacid salts. Only in zinc diacid salts.
1560 Na salts 1579, 1542 Ca salts 1551, 1532 Zn salts, C8, C9 1540 (br) Zn salts C10-C24	1563 Na 1581, 1544 Ca 1551, 1537 Zn	1575 Na 1581, 1542 Ca 1556, 1534 Zn	*v_as_*COO^-^	Singlet for Na and higher Zn salts. Doublet for Ca and lower Zn salts.
1472–1459 Na salts 1473 Ca salts 1467–1464, Zn salts	1463, 14447, 1432, Na 1468, 1455, 1431, 1411 Ca 1467, 1460, 1450 Zn	1463, 1405 Na 1468, 1434, 1420 Ca 1468, 1456 Zn	*δ*CH_2_ scissoring	Single peak, a stable frequency for Ca and higher monoacid Zn salts. Four- or three-fold structuring in diacid salts.
- 1458 Zn salts	n.a.	n.a.	*δ_as_*CH_3_	Overlapped in Na salts; undetectable in Ca salts. Detectable as unresolved shoulder in Zn lower salts.
1423 Na salts 1433, 1411 Ca salts 1410, 1399 Zn salts C8, C9 1398 Zn salts C10-C24	1416 Na 1436, 1405 Ca 1412, 1399 Zn	1434 Na 1431, 1411 Ca 1407, 1398 Zn	*v_s_*COO^-^	Singlet for Na and higher Zn salts. Doublet for Ca and lower Zn salts.
-	n.a.		*δ_s_*CH_3_ (‘umbrella’ vibration)	Undetectable in most monoacid salts
1400–1351 Na salts 1380–1287 Ca salts 1375–1260 Zn salts	1356 Na 1359, 1332 Ca 1366 Zn	1339 Na 1343, 1318 Ca 1353 Zn	*τ*CH2	Progressions of 4 or 5 sub-bands in monoacid salts. Singlet or doublet in diacid salts.
1341–1180 Na salts 1278–1190 Ca salts 1250–1045 Zn salts	1293, 1200 Na 1293, 1261, 1247, 1217, 1201 Ca 1303, 1207 Zn	1316, 1288, 1273, 1253, 1233, 1195 Na 1286, 1251, 1200 Ca 1285, 1243 Zn	*w*CH2	Progressions following the n/2 pattern (n = total carbon atoms) in Na and Ca salts. Sub-bands in Zn salts.
1185–1100 Na 1114 Ca 1067–1105 Zn	1100	1100	Unassigned	Weak single band; up-shifting with longer carbon chains in monoacid Zn salts. Single band; not observed in Na and Zn C8di salts.
855–849			*δ*CH_3_ + vC-C.	Not observed in monoacid zinc and diacid soaps.
725–717, 698 721 Ca salts 747, 723 Zn salts			*ρ*CH2	Doublet for Na and Zn salts; single peak for Ca salts.

*v*: stretching vibration; *vs*: symmetric stretching; *v_as_*: antisymmetric stretching; *δ*: bending vibration; *δ_s_*: symmetric bending; *δ_as_*: antisymmetric bending; *δ*_ip_: in-plane bending; *δ*_oop_: out-of-plane bending; *τ*: twisting vibration; *ρ*: rocking vibration; *w*: wagging vibration.

## Data Availability

The data presented in this study are available on request from the corresponding author.

## Supplementary Materials

The following are available online, Figure S1: Infrared zoomed-in region (1570–1000 cm^−1^ of crystalline (at room temperature)) of even-numbered monoacids: decanoic (C10:0), dodecanoic (C12:0), tetradecanoic (C14:0), hexadecanoic (C16:0), octadecanoic (C18:0), and tetracosanoic (C24:0). Figure S2: Deconvolution and peak fitting of the vC‒H band region of (a) octanoic (C8:0), (b) decanoic (C10:0), and (c) octadecanoic (C18:0) acids. Figure S3: Infrared spectrum of palmitic acid (C16:0) (a) as purchased from the vendor, and (b) after a heating (melting)-annealing cycle. Insets: (i) CH2 wagging region; (ii) CH2 rocking region.

## Abbreviations and Symbols

FA: fatty acids; FAMS: fatty acid metal salts (or metal soaps); FAE: fatty acid esters; FTIR: Fourier Transform Infrared Spectroscopy; C8:0–C24:0 saturated octanoic-tetracosanoic fatty acids; signifies total carbon number; C8di, C9di: octanedioic and nonanedioic acids; Δv: the difference between peak maxima between antisymmetric and symmetric carboxylate absorptions in metal soaps; sFA: saturated fatty acids.
