# Supplementary material for: Fatty Acids and Their Metal Salts: A Review of Their Infrared Spectra in Light of Their Presence in Cultural Heritage
Source: Molecules. 2021 Oct 3;26(19):6005. doi: 10.3390/molecules26196005 (PMC8512802; doi:10.3390/molecules26196005)
Supplement: Supplementary file 1 [file molecules-26-06005-s001.zip › molecules-1393790-supplementary.pdf]

Fatty Acids and Their Metal Salts: A Review of Their Infrared Spectra in Light of Their Presence in Cultural Heritage

Anna Filopoulou, Sophia Vlachou and Stamatis C. Boyatzis *

Department of Conservation of Antiquities & Works of Art, University of West Attica, 12243 Egaleo, Greece

Supplementary Material: Figures S1–S4

Figure S1

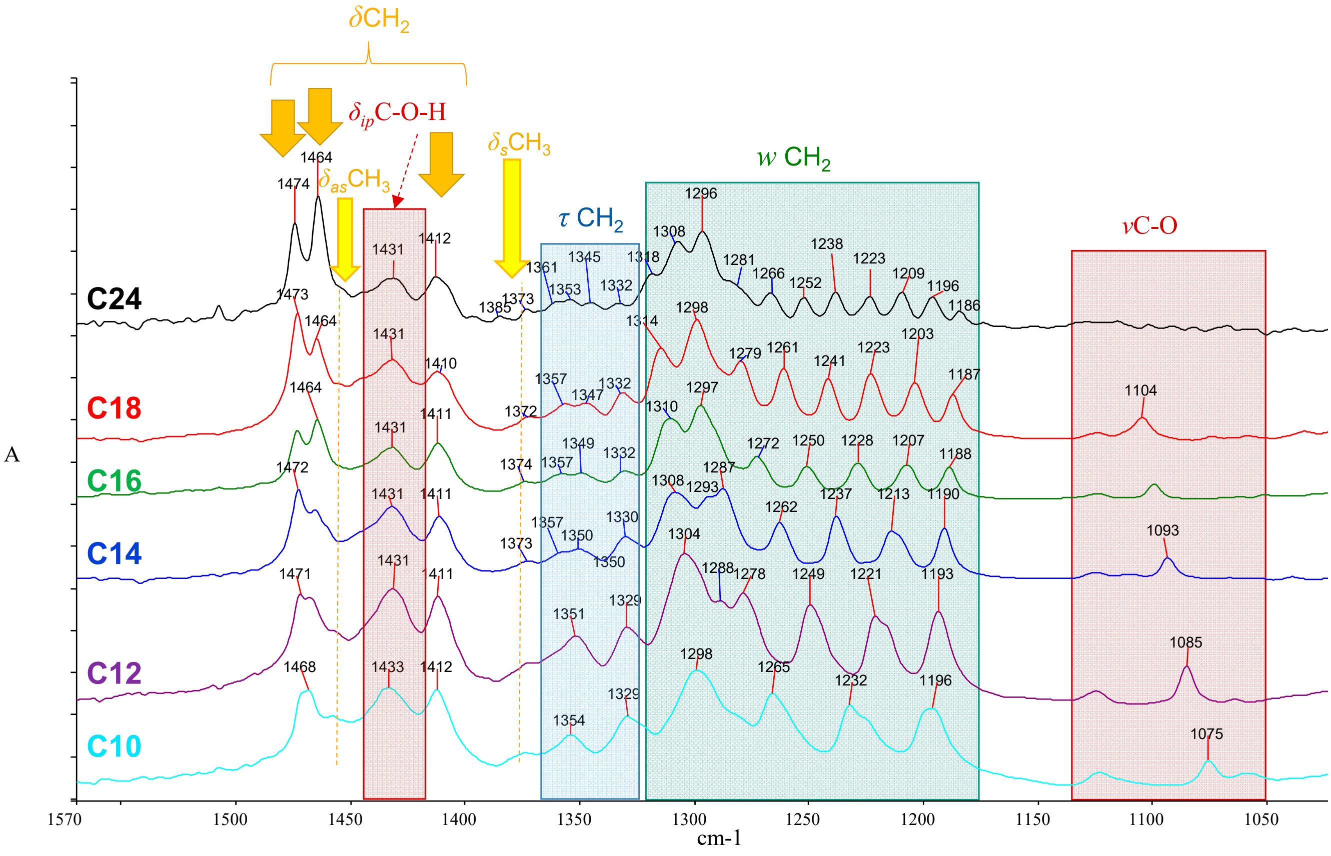

Figure S1. Infrared zoomed-in region (1570 – 1000 cm^{-1} of crystalline (at room temperature) even-numbered monoacids: decanoic (C10:0), dodecanoic (C12:0), tetradecanoic (C14:0), hexadecanoic (C16:0), octadecanoic (C18:0), tetracosanoic (C24:0).

Figure S2

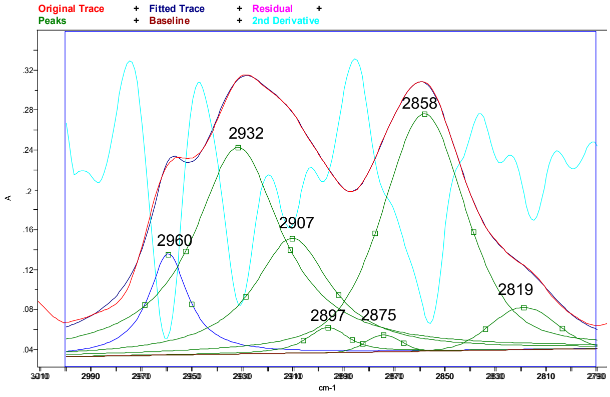

(a)

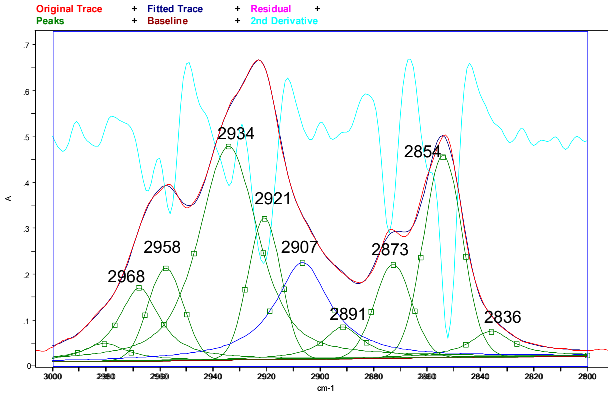

(b)

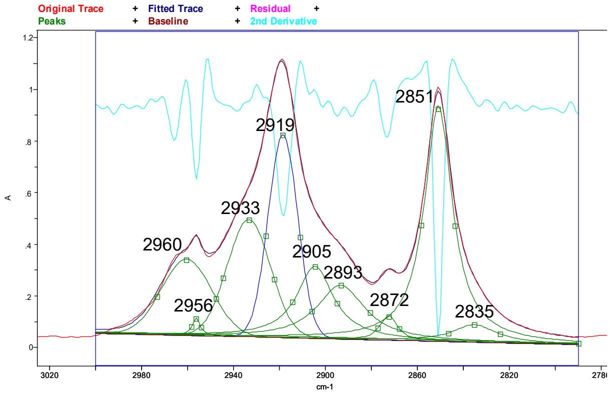

(c)

Figure S2. Deconvolution and peak fitting of the *v*C-H band region of (**a**) octanoic (C8:0), (**b**) decanoic (C10:0) and (**c**) octadecanoic (C18:0) acids.

Figure S3

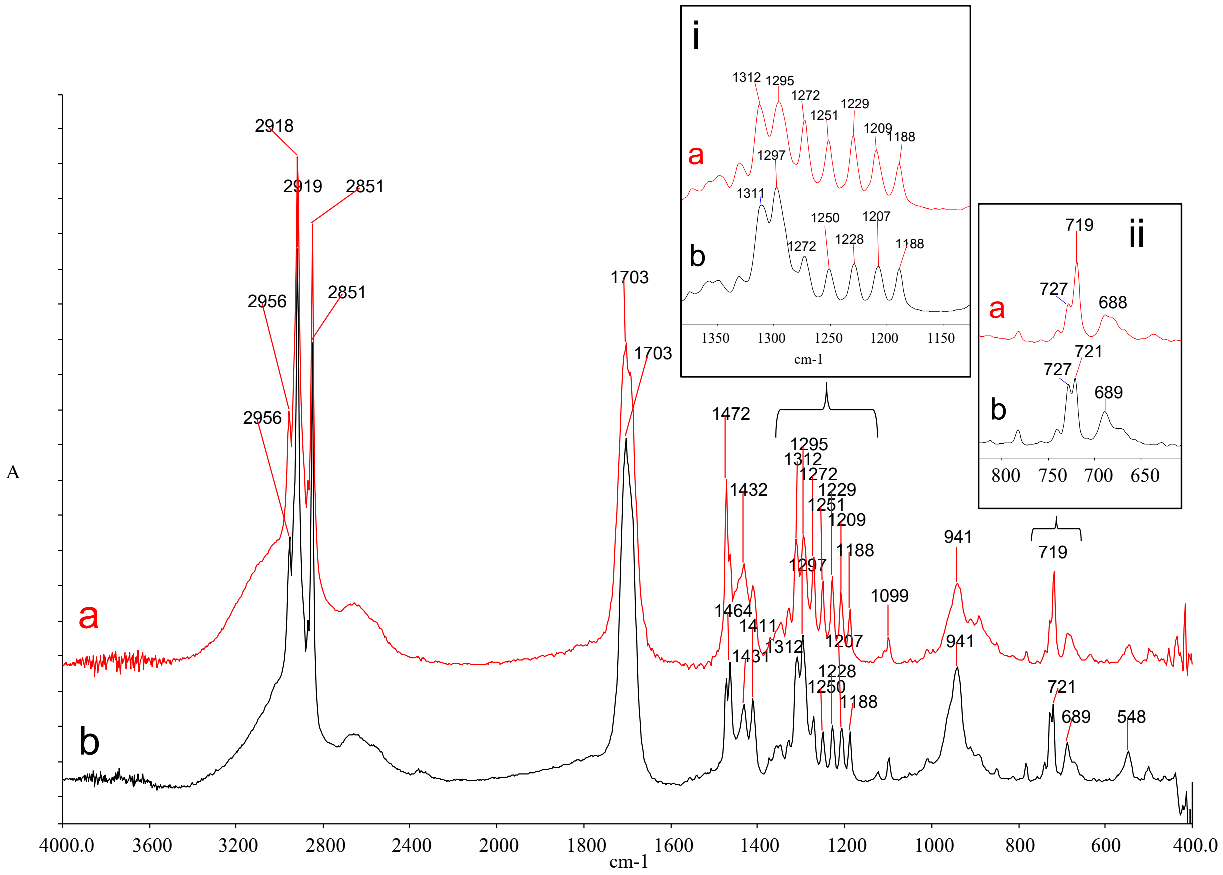

Figure S3. Infrared spectrum of palmitic acid (C16:0) (a) as purchased from vendor, and (b) after a heating (melting) – annealing cycle. Insets: (i) CH_2 wagging region; (ii) CH_2 rocking region

Figure S4

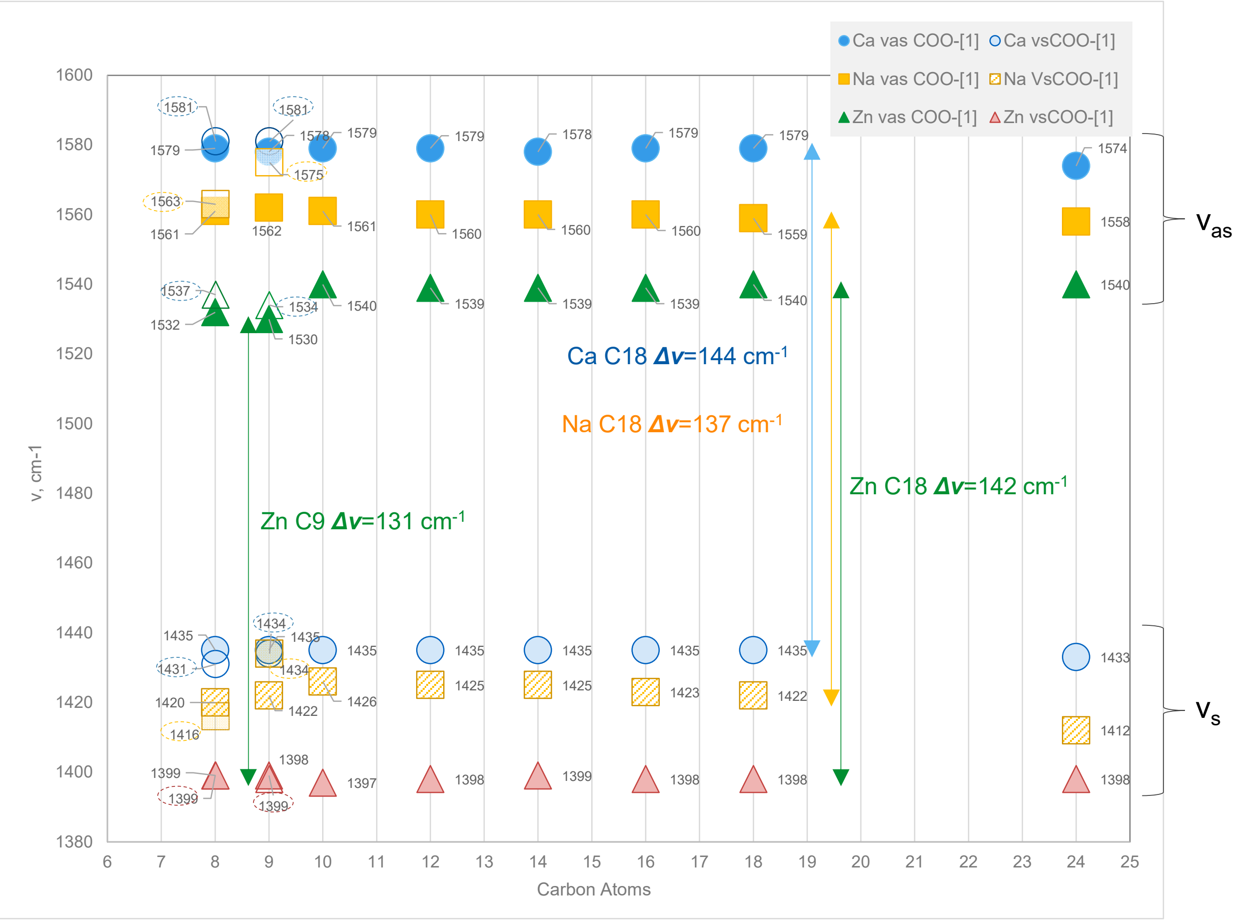

Figure S4. Trends of antisymmetric (v_{as}) and symmetric (v_s) carboxylate stretching maxima of monoacid and diacid metal salts. In cases where bands are split, average values were considered. The Na, Ca and Zn salts of C8di and C9di are shown with open symbols and their maxima labels are circled. Values from spectra in Figures 4, 5, and 7 of main text.
